# 

**Published:** 1894-11-03

**Authors:** 


CONTENTS.

**The Senile Heart** - 73
**The Drug Treatment of Consumption** - 74
**On Toxic Neutralisations.**—By Sir Benjamin W. Richardson, M.D., F.R.S.—III. Physiological Modification, or Catalysis - 75
**The Field of Science** - 76
**Medical Progress and Hospital Clinics:**
Disease of the Heart and Vascular System (*continued*) - 79
Progress in Surgery.—Surgery of the Pancreas and Spleen - 80
Periscope of Dermatology - 81
New Drugs and Preparations - 83
New Appliances and Things Medical - 84
**Annotations.**—Does Baking Sterilise a Loaf?—Milk Diet in Bright's Disease—Doctors and Relieving Officers - 77
**Notes and News** - 78
**Editor's Letter Box** - 84
**The Institutional Workshop:**
Hospital Constuction.—Nurses' Home at Burmantofts (Leeds Union Infirmary—The New Craig House Asylum, Edinburgh - 85
Hospitals in Australia - 86
**Scraps and Gleanings** - 88
**The Book World of Medicine and Science** - 89
**The Student of Medicine** - 89

EXTRA SUPPLEMENT.—THE NURSING MIRROR.

**News from the Nursing World.**—Our Christmas Competitions—The Trained Nurses' Club—Cholera and Common Sense—Addenbrooke's Hospital Cambridge — Fleetwood Cottage Hospital—Women Students—Nursing at Jedburgh—Very Helpless—Sisters at Malta—Civil Hospital, St. Helena—Nurse Bone-Setters—Poona—The Littleborough Nurse—Kimberley Hospital Nursing—Short Items - xxix

**Notes for Nurses on Antiseptic Surgery.**—By William Horrocks, M.B., F.R.C.S. IV.—Preparation for Operation - xxxi

**The Poor of Spitalfields** - xxxi

**Nursing in Germany** - xxxii

**The Royal British Nurses' Association** - xxxii

**Presentations—Appoin ments** - xxxii

**Everybody's Opinion—Nursing in the U.S.A.** - xxxii

**The Duchess of Albany** - xxxiii

**Asylum News and Nursing** - xxxiv

**Male Nurses' Temperance Co-operation** - xxxiv

**Notes and Queries** - xxxiv

